# Effect of Process-Based Multi-Task Cognitive Training Program on Executive Function in Older Adults With Mild Cognitive Impairment: Study Rationale and Protocol Design for a Randomized Controlled Trial

**DOI:** 10.3389/fpsyt.2020.00655

**Published:** 2020-07-23

**Authors:** Xia Zhao, Lina Wang, Chenxi Ge, Xiaoshen Liu, Mei Chen, Chen Zhang

**Affiliations:** ^1^ School of Medicine, Huzhou University, Huzhou Centre Hospital, Huzhou, China; ^2^ Department of Nursing, Huzhou Rehabilitation Hospital, Huzhou, China; ^3^ Department of General Medicine, Community Health Service Center of Renhuangshan, Huzhou, China

**Keywords:** mild cognitive impairment, process-based training, executive function, cognitive assessment, transfer effects

## Abstract

**Introduction:**

Recent research from both human and animal studies confirms that cognitive training gains a considerable effect on multiple cognitive domains in older adults with mild cognitive impairment. Previous studies have yet paid scant attention to executive function training. Little is known about whether this specific benefit translates to maintaining long-term effectiveness and transfer effects are. This study is designed as an effort to address this issue.

**Objective:**

The program aimed to evaluate the effect of process-based multi-task cognitive training on executive function and further explore its long-term effects and transfer effects in older adults with MCI. Furthermore, we will explore the neural correlates latent the changed performances underlying the cognitive intervention.

**Methods:**

This program is a single-blinded, randomized, prospective clinical trial to test the effect of process-based multi-task cognitive training in older adults with MCI. Ninety participants with MCI will be recruited and randomly assigned to the cognitive training group (n=45) and the wait-list control group (n=45). The cognitive training group will receive 10 weeks of process-based multi-task cognitive training and health education twice a week, at 40~60 min per session. While the wait-list control group will only receive 10 weeks of health education during the research period. The effect is measured using the executive function, neuropsychological assessment performance and related brain activity assessed with electroencephalogram parameters (slowness and complexity of the EEG) at baseline, after 10 weeks of training, and a 3-month follow-up.

**Results:**

The study is currently ongoing. Recruitment began in March 2019 and will conclude at the end of 2020. Effects of the process-based multi-task cognitive training on executive function in older adults with MCI will be described in intention-to-treat analysis and protocol set principle. We will also explore the potential long-term effects and transfer effects.

**Discussion:**

If a process-based multi-task cognitive training program results in positive changes to executive function in older adults with MCI, this might provide a viable and potential approach to delay the cognitive decline.

**Clinical Trial Registration:** ChiCTR1900020585. Registered on January 09, 2019. http://www.chictr.org.cn/showproj.aspx?proj=34664.

## Highlights

1. This paper describes the process-based multi-task cognitive training program study, an implementation study to improve the effect on executive function in community elderly with mild cognitive impairment in China. This is the first nurse-led cognitive training program conducted in the community of Zhejiang Province.
2. Previous studies have yet paid scant attention to executive function training. The program adapts the maximum of the optimal training volume to explore whether adequate training could improve executive function.
3. It provides an example of a more cost-effective and feasible cognitive training method and uses dynamic and hierarchical tasks to implement efficiency and suitable.

## Background

Mild cognitive impairment (MCI) represents an intermediate state between normal or healthy ageing and early-stage dementia (1). The annual progression rate from MCI to AD or other types of dementia is 10%~59.4% (2–4), while the proportion from healthy elderly to AD is only 1%~3% (5, 6). Practice guidelines and meta-analysis reported that 14.4%~55.6% of those with MCI revert to normal cognition (NC) (4, 5, 7). The reversion from MCI to NC maybe benefit from appropriate cognition management/intervention (8). Therefore, the stage of MCI provides a critical window of opportunity for the prevention and treatment of dementia.

Although memory impairment is a major manifestation of MCI, preclinical deficits in executive function are widely observed in older adults with MCI (9, 10). Seo pointed out that executive function has been shown to interact with memory function (10). More importantly, the executive function is a crucial predictive factor in dementia prognosis (11). Older adults with MCI with lower executive function demonstrated more often develop into AD after 1 year than those with higher executive function (12). Recently, a growing body of researches on cognitive training has focused on executive dysfunction and found that it could improve executive function performance (13–15). The improvements in executive function are potentially extremely relevant with daily living capacities of older adults (11, 16). Therefore, executive function is considered to be a crucial cognitive domain of the prevention and treatment of MCI.

Cognitive training as a means to counteract cognition decline with high practical operability and no side effects, and has demonstrated its benefits on cognitive function for the older adults with MCI (17–19). In 2017, a review commissioned by the American Academy of Neurology (AAN) recommended that cognitive training may be provided with a beneficial practice in improving measures of cognitive function in older adults with MCI (4). Meanwhile, older adults with MCI in early-stage benefit from neural reorganization, which were induced by cognitive training targeting at executive function (18, 20). Neuroimaging findings also have indicated that cognitive training focus on executive function for normal elderly has been associated with increased neurocognitive activation and cognitive control network connectivity (e.g., frontoparietal network, occipito-temporal regions) (21, 22). Therefore, executive function as a successful compensatory mechanism for cognition improvement could be significant (19, 20).

However, some systematic reviews and RCT studies have reported several limitations frequently occurred in the cognitive training, including an emphasis on single-modality training tasks (23), failure to adjust the cognitive training tasks and the level of difficulty on the training tasks throughout the process of cognitive training (24), and transfer effects of cognitive training to other untrained cognitive domain can be little known (15). Besides, studies of process-based executive function training revealed that more considerable training-related benefits in older adults than in younger adults (25, 26). However, previous process-based cognitive training studies in older adults with MCI did not put sufficient training on the executive function (18, 19).

It remains not entirely clear how cognitive training related mechanisms affect executive function in older adults with MCI. Electroencephalography analysis provides a good indication of the neurological integrity of the central nervous system (27), which would increase our insight into the mechanisms of any potential effects of cognitive training targeting at executive function (28).

Given that the limitations of cognitive training for older adults with MCI reported by previous researches, we developed a process-based multi-tasks cognitive training program based on the process model (29, 30),****which was targeted to improve the executive function of older adults with MCI. The primary aim of this study is to evaluate whether the process-based multi-task cognitive training program would provide better results on executive function in older adults with MCI. The secondary aim is to explore whether potential training effects will be able to generalize to other untrained cognitive domains (transfer effects), and those effects will be maintained effectiveness over 3 months of follow-up (maintaining effect). The tertiary aim is to explore the changed performances of brain neural activity underlying the process-based cognitive training targeting at executive function.

## Materials and Methods

### Study Design and Participants

**Figure 1:**
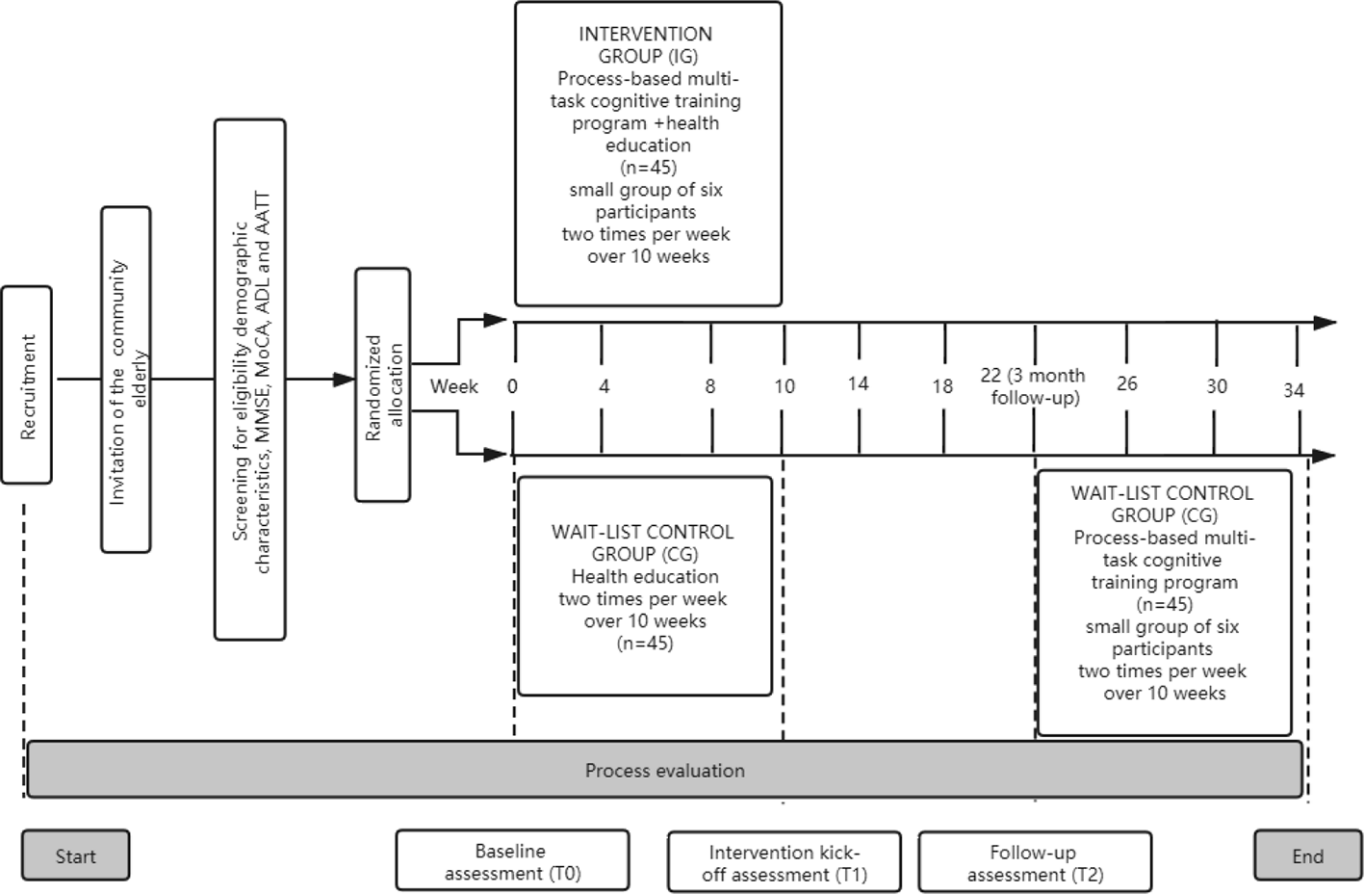
Overview of study procedure..

This study will be a randomized controlled trial (Clinical Trials.gov number: ChiCTR1900020585) with 10 weeks of cognitive training and 3 months of follow-up. The participants will be recruited via advertisements from community healthcare service centre in Huzhou, Zhejiang Province, China. The community healthcare service centre provides comprehensive services, from prevention, screening, and early detection to long-term care that engages communities. The study procedure is shown in **Figure 1**. This program will consist of two arms: the cognitive training group (process-based multi-task cognitive training and health education classes) and the wait-list control group (health education classes). The wait-list group will access to attend the same program after the cognitive training group had completed a 3-month follow-up.

Participants are community-dwelling older adults, both men and women, aged 60 years or older who meet Petersen criteria will be included (31). Participants who meet the following criteria will be allowed to withdraw from this program, having severe medical events that occurred during the training or follow-up period, and should not continue to receive this program, reluctant themselves in training at any time, and a major protocol violation. Detailed inclusion and exclusion criteria are listed in **Table 1**. This study will be carried out following the recommendations of SPIRIT Guidelines with written informed consent from all participants who meet inclusion criteria or their surrogate family members. Ethical approval was approved by the Medical Ethics Committee of the Third People’s Huzhou Hospital of Zhejiang Province (number 2018-030).

**Table 1:** Inclusion and exclusion criteria.

Inclusion criteria	Exclusion criteria
Fulfils the diagnostic criteria of MCI proposed by Petersen (31) Subjective or informant-reported of memory or cognitive decline/complaints Objective memory impairment (or other cognitive functions) inconsistent with age and educational background evaluated by age-adjusted scores at least 1.5 SDs below the mean on the Logical Memory subtest of Chinese version of the Wechsler Memory Scale-Revised (WMS-R) Preserved global cognitive function (MMSE ≥ 24; MoCA <26 when education level >12 years or MoCA <25 when education level≤12 years) Intact activities of daily living Do not meet dementia criteria. Aged 60 years or older Have at least primary school education (>5 years) No significant visual and hearing impairment and color blindness Willingness to participate in entirety of study Capacity to provide written informed consent	History of presence of a neurocognitive disorders (e.g. epilepsy, Parkinson’s disease, head trauma, stroke, mental retardation) and serious medical conditions that can cause brain dysfunction. Take cognitive enhancers, antidepressants in the last month History of presence of severe psychiatric disorders. (e.g., severe depression, active suicidal ideation, anxiety, schizophrenia and alcoholism and drug abuse); Participants in another training related to cognitive function or any investigational drug study at the same time

The calculation of sample size is based on the change in executive function after 10-week, which is the primary outcome variable in this study. A meta-analysis found that significant benefits of cognitive training are superior to the wait-list and the effect size of executive function training used to calculate the sample size is 0.575 [0.093,1.056] (18, 19). Sample size estimation was conducted using statistical software G*power. In the analysis with an independent t-test using a power of 80% and a type 1 error of 5%, the calculated total sample for this study is 78. Considering a 15% dropout rate and maximum to ensure the credibility of the cognitive training, we will recruit 46 participants in each group. Thus, the total required sample size for this study is 92 participants.

### Study Intervention

#### Implementation of the Cognitive Training Program

Participants in the cognitive training group will receive 10 weeks of the process-based multi-task cognitive training program with 60 min group training sessions (a small group of six participants) each given three times per week. This cognitive training program consisted of eleven training tasks in total, with a varying degree of overlap between tasks. The tasks are divided into three categories: 1) warm-up exercise; 2) practice in order; and 3) expanding training (**Table 2**). The task of warm-up exercise lasting 10 min, practice in order and expanding training tasks lasting 50 min. All cognitive tasks are completed within a medical university setting supervision of postgraduate in Master of Nursing Specialist. The program ensures that the ratio of a participant-to-researcher is no greater than 6:1 (6 participants per researcher). Each training task for all nursing specialists was randomly assigned. During the training period, the participant will have an identical training schedule. To balance the social-interaction effect, participants in the cognitive training group participated in the same health education program as the wait-list control group. Participants were reminded via smartphone to complete their training program if missing over 2 weeks. Besides, we develop a training paper record form, and the training record can be checked.

**Table 2:** Tasks of process-based multi-task cognitive training program.

Categories	Tasks	Targeted domain	Difficulty levels/rounds	number of hours	A brief description of the tasks
Warm-up exercise	Recall the names	Memory	0/20	10min	Firstly, introduce yourself one by one (i.e., name, age, occupation). Then, inform participants to remember these names. The participants are asked to write down the names of the other five on white paper according to their memory at the end of cognitive training.
	You act I guess	Working memory	0/1		Firstly, participants were divided into three groups of two. Then, one participant facing the audience uses the most accurate body language to express the meaning of words, while other participants with their backs to the audience are asked to guess those words. Each group is limited to 2 min.
Practice in order	Match the cylinders	Executive function	1/64	50min	Firstly, take any set of socket cylinder socket*, make the cylinder out of the socket, placed near the corresponding socket block, then throw the order of a group of cylinders into confusion. The participants will be required to place each cylinder into appropriate holes in a long wooden socket block with the left-to-right or the right-to-left order. There are sixty-four cylinder socket combinations. With more socket cylinders and change in combination model shown, the difficulty level increases.
	Match the socket apertures		2/64		Firstly, take any set of socket cylinder socket, make the cylinder out of the socket, placed near the corresponding socket block, then throw the order of a group of cylinders into confusion. A wooden cylinder was selected and placed it into the participant’s hand. The participants will be required to find the corresponding position of each cylinder and place it into appropriate holes in a long wooden socket block. With more socket cylinders and changes in the combination model shown, the difficulty level increases.
	Locate the cylinders		3/64		Firstly, take any set of socket cylinder socket on the table, make the cylinder out of the socket, placed socket block on another table. The participants will be required to select suitable cylinders that were shown in the first group and place each cylinder into appropriate holes in a long wooden socket block with the left-to-right or the right-to-left order according to their memory. With more socket cylinders and changes in the combination model shown, the difficulty level increases.
Expanding training	Recall the cylinders		4/64		The participants will be required to complete a task on level 1 (Match the cylinders) with eyes closed. With more socket cylinders and changes in the combination model shown, the difficulty level increases.
	Recall and locate the cylinders		5/64		The participants will be required to complete a task on level 2 (Match the socket apertures) with eyes closed. With more socket cylinders and changes in the combination model shown, the difficulty level increases.
	Recall the sequence of the colored cylinders		6/64		Firstly, take any set of socket cylinder socket with colors ** (Brand Name: DAWONMONTESSORI; Model Number: DW 1002). Then the participants will be required to complete a task on level 1 (Match the cylinders). With more socket cylinders and changes in the combination model shown, the difficulty level increases.
	Stereoscopic cylinders training		7/4		Firstly, take any group of cylinder with colors**. Participants were asked to stack two or more cylinders high according to the card. The card consists of nine squares of the same size. Forty cylinders of different colors and diameters are inside the nine square randomly. The cylinder is not evenly distribute in the square. With more number of stacks, the difficulty level increases.
	Hunt for the colors		8/10		Firstly, take any set of socket cylinder socket with colors (red or green, blue, yellow) are shown. Then the participants will be required to complete a task on level 1 (Match the cylinders) according to cylinder color card (the time of observing card should not exceed 60 s). With more socket cylinders and changes in the combination model shown, the difficulty level increases.

*The first set of cylinders increases in depths and widths, the second set of cylinders only increases in depths with the same widths, the third set of cylinders only increases in widths with the same depths and the last set of cylinders increases in widths but decreases in depths.

**The Cylinders are four sets of wooden cylinders, each a different color with varying widths and depths as same as the cylinder blocks.

#### Intervention Tool

In this study, tasks run on Montessori educational wooden toys—cylinder socket blocks (32). There are 40 wooden cylinders and four sets of wooden socket blocks in total, 10 each per group and each a cylinder with varying widths and depths as same as the corresponding wooden socket block. All cognitive tasks commence with verbal instruction from the trained researcher regarding how to use the socket cylinder and what are the tasks involved. The trained researcher will receive 10 h of training and an intervention manual containing a simplified version of the cognitive training task with illustrations. Participants who fail to complete the task within the set time will be given proper technique guides, but the researchers will not help them complete the tasks.

#### Difficulty Level

The difficulty level, rounds, and progression of cognitive training will be individualized (33). There are 8 levels of difficulty in cognitive training, and 64 same difficulties in each level (except for the task of Stereoscopic cylinders training and Hunt for the colors). The completion time or error frequency of three consecutive training sessions are less than 80% compared with the first attempt, and the training difficulty level will increase. In contrast, the completion time or error frequency of three consecutive training sessions are better than 50% compared with the first attempt, and the training difficulty will decrease. The completion time or error frequency of three consecutive training sessions are 50% to 80% compared with the first attempt; the training difficulty level will increase conversely; the training difficulty level will remain unchanged. Besides, to maintain the challenge and maximize performance, the participants who have not increased the difficulty level for three consecutive weeks will be required to attempt a higher level of difficulty. Each task is presented first at its simplest level, and each subsequent task is a variation of previously mastered skills as the complexity increases.

#### Control Intervention

Participants in the control group are on the waiting list and will receive 10 weeks of health education classes twice a week, at 40~60 min per session. The wait-list group will access to attend the same after the group had completed a 3-month follow-up. The items of health education included the disease knowledge of MCI, physical activity, cognitive activities and social activity. Participants were reminded via smartphone to complete their training program if missing over 2 weeks.

#### Study Procedure

Participants will be enrolled from the nearby community health service centre via open recruitment through paper flyers, online forums, and word-of-mouth. Participants who interested in this program will be invited for an in-person interview to complete a screening questionnaire in the community health service centre, including demographic characteristics, MMSE, MoCA, ADL, and Achromatopsia anomalous trichromatic test (AATT). A trained physician in community health service centre will invite the potential participants to undergo a physical examination and neurologic diagnosis of MCI. Participants who met the eligibility criteria will be assessed with a baseline assessment and then given the EEG evaluation in the 2 days preceding the start of the cognitive training.

After that, according to the random allocation sequence, participants will be randomly assigned to one of two groups with a targeted assignment ratio of 1:1. The random allocation sequence with a block size of four will be generated using a computer randomization list by an independent researcher assistant. The random allocation sequence is sealed in opaque envelopes by an assistant. The cognitive training group will receive cognitive intervention and health education, whereas the wait-list control group will only receive health education during the research period. During the 3 months follow up, the cognitive training group and wait-list control group will receive no interventions. Complete all outcome assessments, participants in the wait-list control group will be permitted to undergo the process-based multi-task cognitive training program. Although it was not initially the plan, the health education manual for mild cognitive impairment will be provided for participants who are not finishing the cognitive training or health education through various means, such as email, public platform, and short messaging service. All outcome measurements will be assessed at baseline, 10 weeks and 3 months follow up. Study assessments and timelines are illustrated in **Table 3**.

**Table 3:** Summary of study assessments and timelines.

Time point	Measurement	Post-allocation					Mode of Administration
		Baseline	Training sessions	10 weeks assessments		Follow-up 3 months	
		T0		T1		T2	
**Enrollment**							
**Eligibility screening**							Paper and pencil
**Informed consent**							Paper and pencil
**Screening**	**General information**						
	Demographic information						Paper and pencil
	**Daily functions**						
	Activities of Daily Living (ADL)						Observation
	**Optic examination**						
	Achromatopsia anomalous trichromatic test						Paper and pencil
	**Neuro psychological examination**						
	Mini–Mental State Examination (MMSE)						Paper and pencil
	Montreal cognitive assessment (MoCA)						Paper and pencil
**Randomized allocation**							
**Intervention**							
**Training tasks**	**↔**					
Warm-up exercise	**√**						Paper and pencil
Practice in order	**√**						Cylinder socket blocks
Expanding training	**√**						
**Assessments**							
**Primary objective**	**Executive function**						
	Trail Making Test A-B	**√**		**√**		**√**	Paper and pencil
	Color-Word Matching Stroop task	**√**		**√**		**√**	Computer
	Purdue pegboard test (Model 32020A)	**√**		**√**		**√**	Device
**Secondary objectives**	**Overall cognitive function**						
	Montreal Cognitive Assessment (MoCA)	**√**		**√**		**√**	Paper and pencil
	**Psychomotor speed**						
	Finger Tapping test	**√**		**√**		**√**	Device
	Reaction Time test (EP203)	**√**		**√**		**√**	Device
	**Transfer effects**						
	Raven’s Advanced Progressive Matrices	**√**		**√**		**√**	Computer
	Digit span test	**√**		**√**		**√**	Paper and pencil
	**Neuroimaging assessments**						
	Electroencephalogram	**√**		**√**		**√**	Computer

### Outcome Measurement

Data evaluators and training practitioners are blinded to group assignments. All outcome measures are assessed at baseline, immediately after 10 weeks of training, and again at follow-up 3 months after the end of cognitive training. Three trained research assistants, with a minimum of 2 years’ experience conducting mental health nursing and neuropsychological assessments, conducted a set of outcome measures. A more detailed account of study assessments and timelines are illustrated in **Table 3**.

The primary outcome measure is the composite z score executive function, which will be accessed by Trail Making Test A-B (TMT A-B), Stroop test (using software, E-Prime 2.0.10.42), and Purdue pegboard test (PPT, Lafayette Instrument Model 32020A). The selection of cognitive evaluation measures, directly relating to the target cognitive field of training, can improve cognitive training power, efficacy and increase accuracy (34, 35). Furthermore, previous studies have shown that the use of TMT A-B, Stroop tests, and PPT as executive function outcome measures in clinical trials of MCI (36, 37). We will calculate z-score composites from normalized raw data for each item and its score as a sum of all domains.

The secondary outcome measures include neuropsychological assessments, transfer effect and electroencephalogram acquisition. Neuropsychological assessments are as follows: (1) the composite z score for the overall cognitive function, including the questionnaire of MoCA (38); (2) the Psychomotor speed, which will be accessed by Finger Tapping Test (37) and Reaction Time test (39). The transfer effects measure consist of fluid reasoning and working memory. Two tasks will be used to measure working memory: digit span forward and backward from the Wechsler Adult Intelligence Scale-Revised. Raven’s Advanced Progressive Matrices (RAPM) will be used to assess fluid reasoning. The electroencephalogram data will be recorded and collected using the Emotiv EPOC® Headset (EMO-EPO-BT9X-03) with 14 channels mobile (AF3, F7, F3, FC5, T7, P7, O1, O2, P8, T8, FC6, F4, F8, and AF4) and two references (CMS/DRL references at P3/P4; left/right mastoid process alternative). The parameters are as follows: the sampling rate is 128 Hz, the resolution is 14 bits with 1 LSB (= 0.51μV), and bandwidth is 0.16–43Hz (digital notch filters at 50Hz and 60Hz). The recording time will last approximately 5 min. The participants will be required to close and open their eyes for 30 s, respectively. During data acquisition time, the participants will be told not to move their hands and other parts of the body (40).

### Statistical Analysis

#### Pre-Processing of Electroencephalogram Data

The actual electroencephalogram signals are recorded using a six-channel configuration at F7, F8, T7, T8, O1, and O2 (Emotiv EPOC® Headset -EMO-EPO-BT9X-03). Lakshmi et al. (41) pointed out that the acquired brain signals are contaminated by noise and artefacts (e.g., eye blinks, eye movements and heartbeat). Besides, interference of muscle movements and power lines are also mingled with brain signals (42). Meanwhile, artefact avoidance is an effective preventive method to minimize artefacts by instructing participants to try to avoid unnecessary blinks, hand/body movements. Therefore, it is necessary to remove artefacts from EEG signals by filtering and feature extraction process.

At first, wavelet denoising is used to preprocess the raw data. By eliminating high frequency, the wavelet method is performed to obtain the finer signal and enhance the effect of noise removal of artefacts (43). Then, a sixth-order band-pass filter with cut-off frequencies of 3 Hz and 13 Hz used in this study to filter the denoised EEG signals. Its purpose is to filter the electromyogram artefacts that tend to remove the unimportant information from high-frequency bands and electrocardiogram that tend to dominate the low-frequency. The next process is feature extraction. Previous studies suggested that principal component analysis is an efficient method the feature extraction (44). Finally, the results of the feature extraction principal component analysis then processed to obtain electroencephalogram spectra of normal condition.

#### Statistical Considerations

The primary and secondary outcome measures were analyzed using data from the intention-to-treat. Moreover, the protocol set principle was defined as those participants or the participants who completed at least 80% adherence to the program initially formulated and assessments all the required will be included. We will compare the characteristics of the completed participants versus dropout ones. Sensitivity analyses of completed participants will be further performed to explore the potential for dropout participants, and the results will be categorized as bias. Listwise deletion will be adopted to estimate treatment effects and sensitivity of the cluster dropout if missing values satisfy the criteria of the losing data entirely at random; otherwise, multiple imputation data strategies will be adopted.

Data processing and statistical analysis will be conducted using SPSS 21.0 and MatLab (The Mathworks Inc. 2016). Descriptive statistics will be reported as frequencies for categorical variables and means ± standard deviations for continuous variables. The adoption mainly the method of exploratory analysis to examine the distribution state of data. The proportionality of baseline data for both groups is compared by the non-parameters Wilcoxon test, chi-square test, and t-test. Regarding the primary outcome, a repeated ANOVA test will be used to evaluate the differences of changes and long-term effect in outcome measurement index performance in the cognitive training group and the wait-list control group, within-group over time, and interaction effects. The simple effect analysis will be added when there is an interaction between intervention factors and time factors. Secondary outcomes will be analyzed using similar statistical methods. The mediating effect model was used to test the transfer effect of cognitive training on untrained cognitive domains, with cognitive training as the mediator. A significant statistical will be considered as P < 0.05 for all comparisons. The effect size (r) was calculated using Cohen’s d to estimate the between-group effect sizes.

## Discussion

Mild cognitive impairment is a current society challenge. Cognitive training has a positive finding in specific cognitive functions with the possibility of greater benefits in individuals with MCI. A meta-analysis study has shown that cognitive training is a practical approach for protecting against cognitive decline (19). Despite this evidence, the alleged positive effects of cognitive training on cognition are yet to reach an agreement. Questions about the relationship between latent the changed performances and neural correlates remain unclear. One primary challenge in implementing cognitive training in the community is the lack of nurse-led cognitive training programs. Given community health care resources in China, only a few large cities can provide professional cognitive trainers and mental health therapists (33). In this study, the process-based multi-task cognitive training program is a randomized controlled trial designed based on a process model. It aims to assess the efficacy of executive function, cognitive function and long-term effect of transfer effects in the community elderly with MCI and determine related neural correlates.

The process-based cognitive training targets more general processing functions (e.g. executive function) (45, 46) and were less affected by the brain or cognitive reserve (25). A study has suggested that process-based cognitive training is feasible to improve executive function and reveal the possibilities of broader generalizations of training effects in healthy older adults (13), as well as in older adults with MCI (47). Sandberg et al. (13) reported that 5 weeks of executive process training sessions significantly enhanced the executive function of older adults with MCI. Previous studies of cognitive training in the elderly with MCI did not place sufficient attention and training on the executive function (20, 23). American cognitive intervention database on ageing (ACTIVE) summarizes cognitive intervention studies that have reported positive results worldwide since 2001 suggested that the optimal training duration and the dose of cognitive training are 6~10 weeks and 40~75 min each given 1~2 times per week respectively (48). Therefore, we adapt to the maximum of the optimal training volume to explore whether adequate training could improve executive function.

It is crucial to recognize the longevity of training gain and transfer effects accurately as Sandberg reported (13). Previous study suggests that the training gains are maintained over 18 months after completion of executive process training (49). Lustig thought that cognitive training for short periods followed by reassessments might be a practical method for maintaining training benefits to minimize the burden for participants (50). Study about cognitive training on working memory indicates that the effects of cognitive training on overall cognitive function and working memory of MCI could be exerted long-term effects even in the absence of cognitive training during follow-up (15). Besides, lacking training generalization may constitute a significant obstacle to the effectiveness of cognitive training (51). The result of Sandberg et al. also stated that executive process training has the greatest effect on the transfer tasks (untrained cognitive domains), which have a substantial process overlap with the trained tasks (49). The older adult exhibit a similar magnitude of transfer as do the young after process-based cognitive training (50). However, transfer to the untrained cognitive domain in the older adults with MCI has been found infrequently and fewer study have explored the longevity of such effects. In this training program, we will explore the cognitive training effects, the longevity of training gain and transfer effects accurately.

There are some hypotheses about the neural correlates of changed cognition underlying cognitive training, which is far from conclusive. One hypothesis suggests that if neural overlap implicates functional overlap, there is a potential for transfer of training between different cognitive domains (50). As suggested by Kennedy et al., it is necessary to make a greater understanding of underlying neural correlate (52), which would provide neural parameters for assessing the effects of differing cognitive training on brain health. However, the absence of definitive neural parameters makes it challenging to compare the outputs of different studies. Besides, few cognitive trainings for the elderly with MCI to date has utilized cognitive neuroscience to direct the development of process-specific interventions. We will include Electroencephalogram along with executive function, neuropsychological assessment performance, to provide more evidence for the neural correlates of changed performances underlying cognitive training.

There are some limitations to the current study. First, the sample population in this study may not represent the overall characteristics of individuals with MCI. In this study, older adults with MCI who have colour blindness would be excluded. Thereby the result will be limited in informing the cognitive benefit generalisability. Second, considering the feasibility, we do not set up an active control group, which may compromise the statistical power. Third, the follow-up cognitive training in this study was limited to 3 months because we will provide compensable training for the wait-list control group after the follow-up period. Hence, conclusions regarding the longer follow-up effectiveness of the process-based multi-task cognitive training program could not be drawn. Future study should further explore the above research gaps.

In summary, our cognitive training program, implemented by socket cylinder and use of dynamic and hierarchical tasks (the difficulty increases as the participants’ performance improves), is easy to complete in older adults with MCI. This use dynamic and hierarchical tasks and multi-modal testing (paper-pencil, computer) will not only provide reliable data on cognitive training and transfer effects but also assure compliance and motivation of participants. The training program is valid, and the effects of training can be sustained will play a vital role in the application of cognitive training in the elderly with MCI. If proven effective, the results of this protocol will be relevant to community health workers to adopt a more cost-effective and feasible cognitive training method for improving the cognitive status, executive function, psychomotor speed, and non-trained cognitive domains.

## Trial Status

The study is currently ongoing. Recruitment began in March 2019 and will conclude at the end of 2020.

## Data Management

During the assessment, all the outcome measures will be recorded by pen and paper. Date collection and informed consent signed will be conducted in a single room and stored in a file cabinet with the password. Only study investigators and related study assistants will have access to the data for analysis. The raw data was entered manually by a researcher assistant and checked independently by two research assistants.

## Risk Analysis

None of the published cognitive training trials reviewed was found adverse effects, and no evidence engaging in these cognitive activities has a negative effect.

## Ethics Statement

The studies involving human participants were reviewed and approved by Ethics approval and consent to participate in the Medical Ethics Committee of the Third People’s Huzhou Hospital of Zhejiang Province has approved and regulates the ethical execution of this research (number 2018-030). The patients/participants provided their written informed consent to participate in this study.

## Author Contributions

All authors contributed to the design and drafting of the manuscript. LW: research concept, research design, and critical revision of the manuscript for intellectual content. XZ: drafting the first version of the manuscript and submit the manuscript for publication. CG: substantially revise and update the protocol prior to initiating the project. XL and MC: assist with statistical analytic planning. CZ: perform a physical examination and neurologic diagnosis for the participants. All authors contributed to the article and approved the submitted version.

## Funding

This work was supported by the National Natural Science Foundation of China (NO.71704053), China Scholarship Council Foundation (NO.201908330251), the Zhejiang provincial Natural Science Foundation of China (NO. LQ17G030002) and the Zhejiang Provincial College Students Scientific and Technological Innovation Activities (2019R431044). The funding bodies did not participate neither in the design of the study nor in the data analysis and manuscript elaboration.

## Conflict of Interest

The authors declare that the research was conducted in the absence of any commercial or financial relationships that could be construed as a potential conflict of interest.

## Abbreviations

MCI, Mild cognitive impairment; MMSE, Mini-mental state examination; TMT A-B, Trail Making Test A-B; PPT, Purdue pegboard test; MoCA, Montreal Cognitive Assessment; RAPM, Raven’s Advanced Progressive Matrices; AATT, Achromatopsia anomalous trichromatic test.

## References

[1] Liang JH Shen WT Li JY Qu XY Li J Jia RX The optimal treatment for improving cognitive function in elder people with mild cognitive impairment incorporating Bayesian network meta-analysis and systematic review. Ageing Res Rev (2019) 51:85–96. 10.1016/j.arr.2019.01.009 30682429

[2] Parnetti L Chipi E Salvadori N D’Andrea K Eusebi P Prevalence and risk of progression of preclinical Alzheimer’s disease stages: a systematic review and meta-analysis. Alzheimers Res Ther (2019) 11(1):7. 10.1186/s13195-018-0459-7 30646955 PMC6334406

[3] LoBue C Woon FL Rossetti HC Hynan LS Hart J Cullum CM Traumatic brain injury history and progression from mild cognitive impairment to Alzheimer disease. Neuropsycho-logy (2018) 32(4):401–9. 10.1037/neu0000431 PMC5975979 29809031

[4] Petersen RC Lopez O Armstrong MJ Getchius TSD Ganguli M Gloss D Practice guideline update summary: Mild cognitive impairment: Report of the Guideline Development, Dissemination, and Implementation Subcommittee of the American Academy of Neurology. Neurology (2018) 90(3):126–35. 10.1212/WNL.0000000000004826 PMC5772157 29282327

[5] Gill L Andrew S Vasiliki O Sergi GC Jonathan H David A Dementia prevention, intervention, and care. Lancet (2017) 390:2673–734. 10.1016/S0140-6736(17)31363-6 28735855

[6] Bohlken J Jacob L Kostev K Progression of mild cognitive impairment to dementia in German specialist practices. Dementia (2019) 18(1):380–90. 10.1177/1471301216673919 27758960

[7] Wood H Alzheimer disease: Meta-analysis finds high reversion rate from MCI to normal cognition. Nat Rev Neurol (2016) 12:189. 10.1038/nrneurol.2016.29 26965671

[8] Godinho C Camozzato AL Onyszko D Chaves ML Estimation of the risk of conversion of mild cognitive impairment of Alzheimer type to Alzheimer’s disease in a south Brazilian population-based elderly cohort: the PALA study. Int Psychogeriatr (2012) 24(4):674–81. 10.1017/S1041610211002043 22088617

[9] Kirova AM Bays RB Lagalwar S Working memory and executive function decline across normal aging, mild cognitive impairment, and Alzheimer’s disease. BioMed Res Int (2015) 2015:748212. 10.1155/2015/748212 26550575 PMC4624908

[10] Seo EH Kim H Lee KH Choo IH Altered Executive Function in Pre-Mild Cognitive Impairment. J Alzheimers Dis (2016) 54(3):933–40. 10.3233/JAD-160052 27567814

[11] Mansbach WE Mace RA Predicting Functional Dependence in Mild Cognitive Impairment: Differential Contributions of Memory and Executive Functions. Gerontologist (2019) 59(5):925–35. 10.1093/geront/gny097 30137363

[12] Chang YL Jacobson MW Fennema-Notestine C Hagler D Jennings RG Dale AM Level of executive function inﬂuences verbal memory in amnestic mild cognitive impairment and predicts prefrontal and posterior cingulate thickness. Cereb Cortex (2010) 20(6):1305–13. 10.1093/cercor/bhp192 PMC2912652 19776343

[13] Sandberg P Ro¨nnlund M Nyberg L Neely AS Executive process training in young and old adults. Aging Neuropsychol Cognit (2014) 21(5):577–605. 10.1080/13825585.2013.839777 24148093

[14] Tang Y Xing Y Zhu Z He Y Li F Yang J The effects of 7-week cognitive training in patients with vascular cognitive impairment, no dementia (the Cog-VACCINE study): A randomized controlled trial. Alzheimers Dement (2019) 15(5):605–14. 10.1016/j.jalz.2019.01.009 30894299

[15] Weng W Liang J Xue J Zhu T Jiang Y Wang J The Transfer Effects of Cognitive Training on Working Memory Among Chinese Older Adults With Mild Cognitive Impairment: A Randomized Controlled Trial. Front Aging Neurosci (2019) 11:212. 10.3389/fnagi.2019.00212 31474850 PMC6702334

[16] Karr JE Areshenkoff CN Rast P Garcia-Barrera MA An empirical comparison of the therapeutic benefits of physical exercise and cognitive training on the executive functions of older adults: a meta-analysis of controlled trials. Neuropsychology (2014) 28(6):829–45. 10.1037/neu0000101 24933486

[17] Ngandu T Lehtisalo J Solomon A Levälahti E Ahtiluoto S Antikainen R A 2 year multidomain intervention of diet, exercise, cognitive training, and vascular risk monitoring versus control to prevent cognitive decline in at-risk elderly people (FINGER): a randomised controlled trial. Lancet (2015) 385(9984):2255–63. 10.1016/S0140-6736(15)60461-5 25771249

[18] Sherman DS Mauser J Nuno M Sherzai D The efficacy of cognitive intervention in mild cognitive impairment (MCI): A meta-analysis of outcomes on neuropsychological measures. Neuropsychol Rev (2017) 27(4):440–84. 10.1007/s11065-017-9363-3 PMC5754430 29282641

[19] Hill NT Mowszowski L Naismith SL Chadwick VL Valenzuela M Lampit A Computerized cognitive training in older adults with mild cognitive impairment or dementia: A systematic review and meta-analysis. Am J Psychiatry (2017) 174(4):329–40. 10.1176/appi.ajp.2016.16030360 27838936

[20] Clément F Gauthier S Belleville S Executive functions in mild cognitive impairment: emergence and breakdown of neural plasticity. Cortex (2013) 49(5):1268–79. 10.1016/j.cortex.2012.06.004 22841389

[21] Kim H Chey J Lee S Effects of Multicomponent Training of Cognitive Control on Cognitive Function and Brain Activation in Older Adults. Neurosci Res (2017) 124:8–15. 10.1016/j.neures.2017.05.004 28577979

[22] Breukelaar IA Williams LM Antees C Grieve SM Foster SL Gomes L Cognitive Ability Is Associated With Changes in the Functional Organization of the Cognitive Control Brain Network. Hum Brain Mapp (2018) 39(12):5028–38. 10.1002/hbm.24342 PMC6866537 30136345

[23] Daugherty AM Zwilling C Paul EJ Sherepa N Allen C Arthur FK Multi-modal fitness and cognitive training to enhance fluid intelligence. Intelligence (2018) 66:32–43. 10.1016/j.intell.2017.11.001

[24] Buitenweg JIV van de Ven RM Prinssen S Murre JMJ Ridderinkhof KR Cognitive Flexibility Training: A Large-Scale Multimodal Adaptive Active-Control Intervention Study in Healthy Older Adults. Front Hum Neurosci (2017) 11:529. 10.3389/fnhum.2017.00529 29209183 PMC5701641

[25] Karbach J Kray J How useful is executive control training? Age differences in near and far transfer of task-switching training. Dev Sci (2009) 12(6):978–90. 10.1111/j.14677687.2009.00846.x 19840052

[26] Bherer L Kramer AF Peterson MS Colcombe S Erickson K Becic E Transfer effects in task-set cost and dual-task cost after dual-task training in older and younger adults: further evidence for cognitive plasticity in attentional control in late adulthood. Exp Aging Res (2008) 34(3):188–219. 10.1080/03610730802070068 18568979 PMC2845439

[27] Deursen van JA Vuurman EF Verhey FR Kranen-Mastenbroek van VH Riedel WJ Increased EEG gamma band activity in Alzheimer’s disease and mild cognitive impairment. J Neural Transm (2008) 115(9):1301–11. 10.1007/s00702-008-0083-y PMC2525849 18607528

[28] Abásolo D Hornero R Espino P Escudero J Gómez C Electroencephalogram back- ground activity characterization with approximate entropy and auto mutual information in Alzheimer’s disease patients. Conf Proc IEEE Eng Med Biol Soc (2007) 2007:6192–5. 10.1109/IEMBS.2007.4353769 18003435

[29] Kliegel M Altgassen M Hering A Rose NS A process-model based approach to prospective memory impairment in Parkinson’s disease. Neuropsychologia (2011) 49(8):2166–77. 10.1016/j.neuropsychologia.2011.01.024 21255592

[30] Kliegel M Martin M McDaniel MA Einstien GO Complex prospective memory and executive control of working memory: A process model. Psychol Test Assess Model (2002) 44(2):303–18. 10.1053/ejso.2002.1329

[31] Petersen RC Morris JC Mild cognitive impairment as a clinical entity and treatment target. Arch Neurol (2005) 62(7):1160–3. 10.1001/archneur.62.7.1160 16009779

[32] Camp CJ Judge KS Bye CA Fox KM Bowden J Bell M An intergenerational program for persons with dementia using Montessori methods. Gerontologist (1997) 37(5):688–92. 10.1093/geront/37.5.688 9343920

[33] Zhang H Wang J Sun T Wang Z Lyu X Yu X A randomized controlled trial of combined executive function and memory training on the cognitive and noncognitive function of individuals with mild cognitive impairment: Study rationale and protocol design. Alzheimers Dement (2018) 4:556–64. 10.1016/j.trci.2018.09.004 PMC6205116 30386820

[34] Greenaway MC Hanna SM Lepore SW Smith GE. A Behavioral Rehabilitation Intervention for Amnestic Mild Cognitive Impairment. Am J Alzheimers Dis Other Demen (2008) 23(5):451–61. 10.1177/1533317508320352 PMC2845519 18955724

[35] Kinsella GJ Mullaly E Rand E Ong B Burton C Price S Early intervention for mild cognitive impairment: a randomised controlled trial. J Neurol Neurosurg Psychiatry (2009) 80:730–6. 10.1136/jnnp.2008.148346 19332424

[36] Godefroy O Martinaud O Narme P Joseph PA Mosca C Lhommée E Dysexecutive disorders and their diagnosis: A position paper. Cortex (2018) 109:322–35. 10.1016/j.cortex.2018.09.026 30415091

[37] Jiang H Chen S Wang L Liu X An Investigation of Limbs Exercise as a Treatment in Improving the Psychomotor Speed in Older Adults With Mild Cognitive Impairment. Brain Sci (2019) 9(10):277. 10.3390/brainsci9100277 PMC6827026 31623274

[38] Siqueira GSA Hagemann P de MS Coelho D de S Dos Santos FH Bertolucci PHF Can MoCA and MMSE Be Interchangeable Cognitive Screening Tools? A Systematic Review. Gerontologist (2019) 59(6):e743–63. 10.1093/geront/gny126 30517634

[39] Albinet CT Boucard G Bouquet CA Audiffren M Processing Speed and Executive Functions in Cognitive Aging: How to Disentangle Their Mutual Relationship? Brain Cognit (2012) 79(1):1–11. 10.1016/j.bandc.2012.02.001 22387275

[40] Barham MP Clark GM Hayden MJ Enticott PG Conduit R Lum JAG Acquiring research-grade ERPs on a shoestring budget: A comparison of a modified Emotiv and commercial SynAmps EEG system. Psychophysiology (2017) 54(9):1393–404. 10.1111/psyp.12888 28497557

[41] Lakshmi MR Prasad TV Prakash DVC Survey on EEG signal processing methods. Int J Adv Res Comput Sci Softw Eng (2014) 4(1):84–91.

[42] Teplan M Fundamentals of EEG measurement, Measure. Sci Rev (2002) 2:1–11.

[43] Islam MK Rastegarnia A Yang Z Methods for artifact detection and removal from scalp EEG: A review. Neurophysiol Clin (2016) 46:287–305. 10.1016/j.neucli.2016.07.002 27751622

[44] Turnip A Automatic artifacts removal of EEG signals using robust principal component analysis. IEEE (2014) 331–44. 10.1109/TIME-E.2014.7011641

[45] Karbach J Verhaeghen P Making working memory work: a meta-analysis of executive-control and working memory training in older adults. Psychol Sci (2014) 25(11):2027–37. 10.1177/0956797614548725 PMC4381540 25298292

[46] Gavelin HM Boraxbekk CJ Stenlund T Järvholm LS Neely AS Effects of a process-based cognitive training intervention for patients with stress-related exhaustion. Stress (2015) 18:578–88. 10.3109/10253890.2015.1064892 26305186

[47] Grönholm-Nyman P Can Executive Functions Be Trained in Healthy Older Adults and in Older Adults with Mild Cognitive Impairment? Health and Cognition in Old Age. (2015) 10:233–43 10.1007/978-3-319-06650-9_15

[48] Ross LA Sprague BN Phillips CB O’Connor ML Dodson JE The Impact of Three Cognitive Training Interventions on Older Adults’ Physical Functioning across Five Years. J Aging Health (2018) 30:475–98. 10.1177/0898264316682916 PMC5453841 28553791

[49] Sandberg P Stigsdotter Neely A Long-term effects of executive process training in young and old adults. Neuropsychol Rehabil (2016) 26:761–82. 10.1080/09602011.2015.1108205 26599201

[50] Lustig C Shah P Seidler R Reuter-Lorenz PA Aging, training, and the brain: a review and future directions. Neuropsychol Rev (2009) 19:504–22. 10.1007/s11065-009-9119-9 PMC3005345 19876740

[51] Woods AJ Cohen R Marsiske M Alexander GE Czaja SJ Wu S Augmenting cognitive training in older adults (The ACT Study): Design and Methods of a Phase III tDCS and cognitive training trial. Contemp Clin Trials (2018) 65:19–32. 10.1016/j.cct.2017.11.017 29313802 PMC5803439

[52] Kennedy G Hardman RJ MacPherson H Scholey AB Pipingas A How does exercise reduce the rate of age-associated cognitive decline? A review of potential mechanisms. J Alzheimers Dis (2016) 55:1–18. 10.3233/JAD-160665 27636853

